# Demographic routes to variability and regulation in bird populations

**DOI:** 10.1038/ncomms12001

**Published:** 2016-06-22

**Authors:** Bernt-Erik Sæther, Vidar Grøtan, Steinar Engen, Tim Coulson, Peter R. Grant, Marcel E. Visser, Jon E. Brommer, B. Rosemary Grant, Lars Gustafsson, Ben J. Hatchwell, Kurt Jerstad, Patrik Karell, Hannu Pietiäinen, Alexandre Roulin, Ole W. Røstad, Henri Weimerskirch

**Affiliations:** 1 Department of Biology, Centre for Biodiversity Dynamics, Norwegian University of Science and Technology, NO-7491 Trondheim, Norway; 2 Department of Mathematical Sciences, Centre for Biodiversity Dynamics, Norwegian University of Science and Technology, NO-7491 Trondheim, Norway; 3 Department of Zoology, University of Oxford, South Parks Road, OX1 3PS Oxford, UK; 4 Department of Ecology and Evolutionary Biology, Princeton University, Princeton, New Jersey 08544, USA; 5 Department of Animal Ecology, Netherlands Institute of Ecology (NIOO-KNAW), Wageningen, The Netherlands; 6 Department of Biology, University Hill, University of Turku, FI-02700 Turku, Finland; 7 Department of Animal Ecology, Evolutionary Biology Centre, Uppsala University, Norbyvägen 18D, SE-752 36 Uppsala, Sweden; 8 Department of Animal & Plant Sciences, University of Sheffield, Western Bank, Sheffield S10 2TN, UK; 9 Aurebekksveien 61, NO-4516 Mandal, Norway; 10 Department of Biosciences, Environmental and Marine Biology, Åbo Akademi University, FI-02700 Turku, Finland; 11 Coastal Zone Research Team, Novia University of Applied Sciences, Raseborgsvägen 9, FI-10600 Ekenäs, Finland; 12 Bird Ecology Unit, Department of Biosciences, University of Helsinki, FI-00014 Helsinki, Finland; 13 Department of Ecology and Evolution, Biophore, University of Lausannne, 1024 Lausanne, Switzerland; 14 Department of Ecology and Natural Resource Management, Norwegian University of Life Sciences, NO-1432, Ås, Norway; 15 Centre d'Etudes Biologiques de Chizé, CNRS-UPR 1934, 79360 Villiers en Bois, France

## Abstract

There is large interspecific variation in the magnitude of population fluctuations, even among closely related species. The factors generating this variation are not well understood, primarily because of the challenges of separating the relative impact of variation in population size from fluctuations in the environment. Here, we show using demographic data from 13 bird populations that magnitudes of fluctuations in population size are mainly driven by stochastic fluctuations in the environment. Regulation towards an equilibrium population size occurs through density-dependent mortality. At small population sizes, population dynamics are primarily driven by environment-driven variation in recruitment, whereas close to the carrying capacity *K*, variation in population growth is more strongly influenced by density-dependent mortality of both juveniles and adults. Our results provide evidence for the hypothesis proposed by Lack that population fluctuations in birds arise from temporal variation in the difference between density-independent recruitment and density-dependent mortality during the non-breeding season.

## Abstract (web summary)

Why does population variability differ? Sæther *et al*. show that the magnitude of temporal variation of avian population fluctuations is mainly determined by environmental fluctuations affecting recruitment, whereas regulation of mean population size occurs through density-dependent mortality.

## Graphical abstract

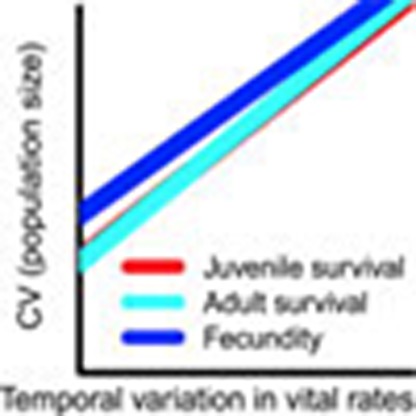

Temporal variation in population size is primarily determined by stochastic fluctuations in the environment and density-dependence^1^,^2^,^3^,^4^,^5^. An important advance in population ecology was provided by May^6^, who showed that even in a stable environment simple population models could produce complicated patterns of population fluctuations, strongly dependent upon the strength and form of density regulation. This theoretical insight spurred extensive analyses of the density-dependence in time series of population fluctuations^2^,^5^ that were aimed to reveal the influence of density-dependent processes on population dynamics. However, generalization of the results from these comparative analyses involving numerous taxa proved difficult because they revealed large differences among species in how changes in population size affect different parts of their life history^7^,^8^,^9^,^10^,^11^. One reason for this is that the sensitivity of population growth rate to changes in a specific vital rate is dependent upon both the species' life history^12^,^13^,^14^,^15^, and how close the population size is to the carrying capacity *K*^15^,^16^. Thus, the impact of environmental perturbations on the growth of the population is consequently affected by the interaction between life history and density-dependence.

One of the few testable hypotheses in population ecology was provided by Lack^17^, who proposed that changes in population size in birds are determined by (i) the density-independent additions of new recruits to the population determined by the available food supply during the breeding season and (ii) density-dependent mortality during the non-breeding season. An important extension of Lack's hypothesis was provided by Ashmole^18^, who suggested, assuming that population regulation occurs during the non-breeding season, that high reproductive rates were favoured by large seasonal fluctuations in food supply^19^. There is some empirical within-species support for this tap-tub model^20^ of population dynamics^17^,^21^, but it is not known whether the relative contributions of these two processes vary predictably across species as a function of their life history.

Here, we test Lack's^4^ hypothesis by using post-breeding, stochastic density-dependent matrix models that allow us to partition the effects of density-dependence and environmental stochasticity on the population growth rate of 13 species of birds. We show that different demographic traits change in a similar way in all species as population sizes approaches *K*. At small population sizes environmental stochasticity primarily affecting variation in fecundity rates is the main driver for changes in population size. This environmentally-driven variation in fecundity is also the major factor affecting interspecific differences in population variability. When population sizes are closer to the carrying capacity *K*, fluctuations in population size are most strongly influenced by temporal variation in survival, determining the strength of population regulation. Lack's simple conceptual model^17^ to explain regulation and limitation of bird populations can consequently provide a general framework for quantitative analyses of population dynamics in fluctuating environments.

## Results

In order to parameterise our models from data we needed detailed, long-term, individual-based life history data from populations where density-dependence was operating (see Methods section; Supplementary Fig. 1). We tested for density-dependence by regressing the change in population size from year *t* to *t*+1 Δ*N*_*t*_ against population size in year *t*^22^. The magnitude of population variability was characterized by the coefficient of variation of the stationary distribution of population sizes around the carrying capacity *K*^5^, which was calculated from the time series of variation in population size (see Methods section). There was nearly an order of magnitude difference in the CVs (standardized in relation to *K*) between species, with similar sized birds exhibiting very different values: CV ranged from 0.077 in the collared flycatcher *Ficedula albicollis* to 0.479 in the medium ground finch *Geospiza fortis*. As is common in birds^7^, the CV was independent of the strength of density-dependence measured as the inverse of the mean return time to the carrying capacity *K*^2^ (Pearson correlation *r*=0.004, *P*=0.83, *n*=13). These statistical analyses of the time series raise questions about how density-dependence and environmental stochasticity (stochastic variation affecting the whole or parts of the population similarly) influence the population dynamics in birds, and why species of very similar size can exhibit such contrasting dynamics. In order to answer these questions we construct and analyze a stochastic, density-dependent matrix model for each species (see Methods section).

**Figure 1:**
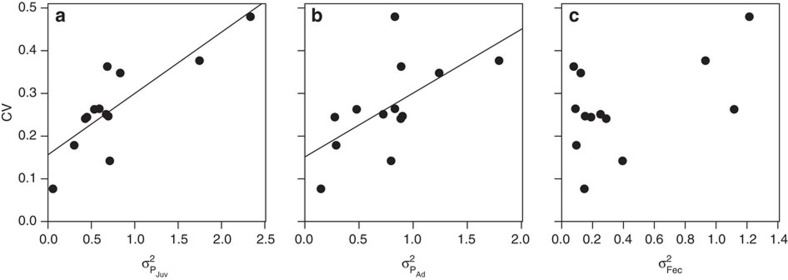
Interspecific differences in population variability in relation to environmental stochasticity in different vital rates. The coefficient of variation in the stationary distribution of population sizes around the carrying capacity *K*, assuming a logistic model of density regulation, in relation to environmental stochasticity in juvenile survival rate (**a**), adult survival rate (**b**) and number of fledglings produced (**c**). The equations for the linear regression lines are *y*=0.14*x*+0.16; *r*^2^=0.68; df=1, 11; *P* < 0.001; *y*=0.14*x*+0.16; *r*^2^=0.41; df=1, 11; *P* <0.01; and *y*=0.13*x*+0.22; *r*^2^=0.26; df=1, 11; *P*=0.073 (not shown) for juvenile survival rate, adult survival rate and fledgling production, respectively.

For all species, the environmental variance in each demographic rate contributed to the environmental stochasticity exhibited in the population dynamics within each species (Fig. 1). When these contributions were compared across species, we found positive associations between the CV and environmental stochasticity in juvenile survival (Fig. 1a), adult survival (Fig. 1b) and fecundity rates (Fig. 1c). The associations were tighter for the survival rates (linear regression analysis *P*=0.0005 and *P*=0.025 for juveniles and adults, respectively) compared to the fecundity rates (linear regression analysis *P*>0.07). On average, environmental stochasticity in each demographic rate was linearly translated into a proportional amount of environmental stochasticity in the population dynamics. However, the environmental stochasticity in each of the demographic rates was not always independent of one another. For example, environmental stochasticity in juvenile survival was positively correlated across species with the environmental noise in fecundity (Pearson correlation *r*=0.71, *P*=0.006, *n*=13) and adult survival (Pearson correlation *r*=0.61, *P*=0.03, *n*=13), while the environmental stochasticity in adult survival was statistically independent of that in fecundity (Pearson correlation *r*=0.26, *P*=0.39, *n*=13). Taken together, these results suggest that environmental stochasticity in all rates can contribute to population fluctuations^12^, but that there was large interspecific variation in the magnitude of these environmental influences. These patterns seem independent of the choice of model for density regulation (Supplementary Note 1; Supplementary Fig. 2; Supplementary Table 1).

**Figure 2:**
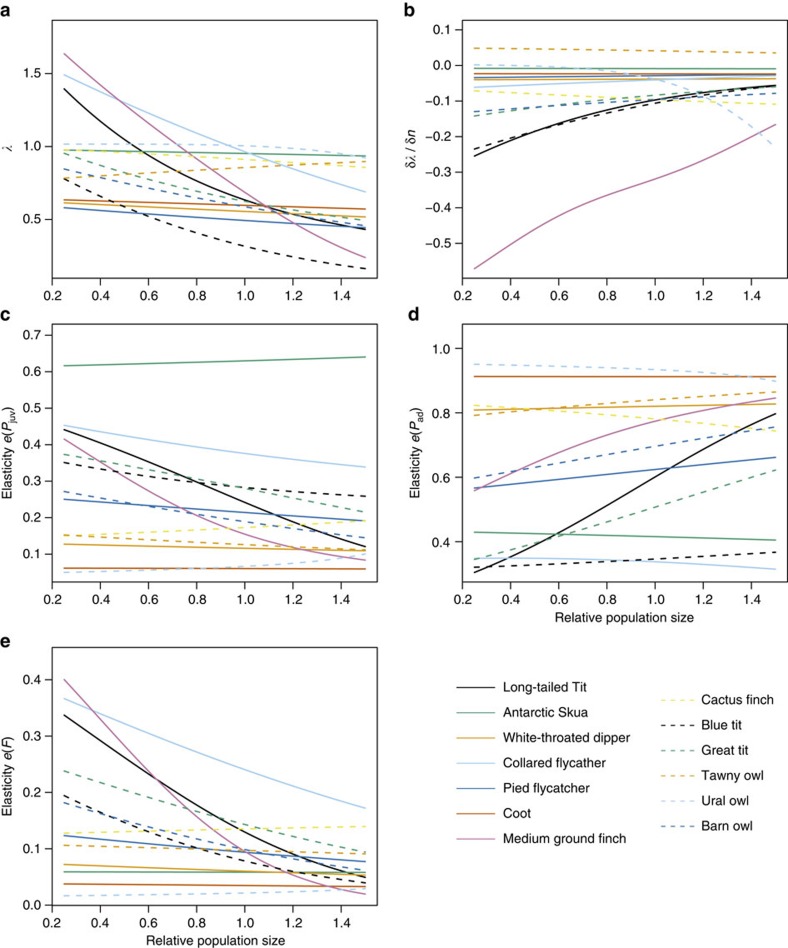
Interspecific differences in the density-dependence of the population growth rate *λ* and the elasticities of different vital traits. The change with population size 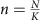, (where the carrying capacity *K* was estimated from the time series of populations fluctuations using a logistic model of density regulation) against the population growth rate *λ* (**a**), the strength of density-dependence in the population growth rate 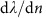 (**b**), and in the elasticity of *λ* to changes in juvenile survival rate (**c**), adult survival rate (**d**) and fecundity rate (**e**).

We next turn to characterizing the contribution of density-dependence to the population dynamics by analyzing each stochastic, density-dependent matrix model, modelling the population growth rate as function of relative population size *n*=*N*/*K*, where *K* was estimated from the time series of fluctuations in population size *N*. There was considerable interspecific variation in both the shape of density-dependence (Fig. 2a) and in its strength (Fig. 2b), measured as the relative change in the population growth rate *λ* calculated from the projection matrix at different relative densities from 0.25*K* to *K*^23^. In all but one species, the population growth rate decreased as population size increased (Fig. 2a; Supplementary Fig. 3). However, the shape of the density-dependence was typically concave-up (Fig. 2b), which meant that the strength of density-dependence typically declined as population size increased. Populations are consequently predicted to converge towards a carrying capacity, but the rate of convergence is fastest at lower densities.

The primary reason that the shape of the density-dependence was concave-up is that the elasticity of fecundity to population size decreased as population size increased in 11 out of 13 species (Fig. 2e). In contrast, there was no consistent cross-species pattern in the elasticity of either juvenile (Fig. 2c) or adult survival (Fig. 2d) with population size. This means that as relative population size increases and the population approaches carrying capacity, then fecundity becomes much less important in determining dynamics than it was at low densities. In contrast, the relative contribution of survival increases. Density-dependence in survival consequently regulates population growth rate.

**Figure 3:**
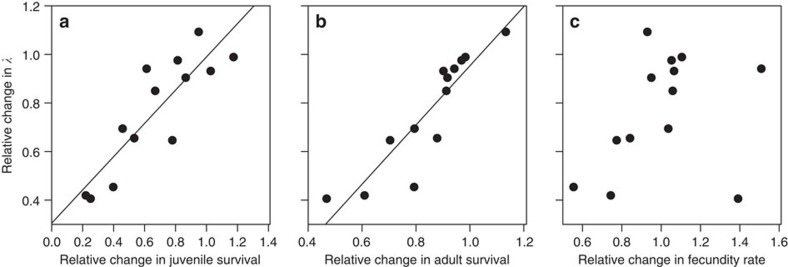
Influence of different vital rates on the density-dependence in the population growth rate *λ*. Interspecific differences in the relative change of *λ* from 0.25*K* to *K* in relation to relative changes in juvenile survival rate (**a**), adult survival rate (**b**) and fecundity rate (**c**) over the same interval of variation in population size. The equations for the linear regression lines are *y*=0.68*x*+0.30; *r*^2^=0.74; df=1, 11; *P*=0.0015; *y*=1.22*x*−0.27; *r*^2^=0.81; df=1, 11; *P* <0.001; and *y*=0.30*x*+0.46; *r*^2^=0.11; df=1, 11; *P*=0.27 (not shown) for juvenile survival rate, adult survival rate and fecundity rate, respectively.

To illustrate this demographic effect of density-dependence further, we calculated the population growth rate and juvenile survival, adult survival and fecundity at 25% of carrying capacity and at carrying capacity. We then calculated the difference between each quantity evaluated at the two densities. By regressing the relative difference in the population growth rate against the relative difference in each demographic rate, we were able to show that there were statistically significant positive associations for juvenile and adult survival, but not for fecundity (Fig. 3, see also Supplementary Fig. 4 for similar results for the loglinear model of density regulation). The most pronounced density-dependent changes occurred in juvenile survival rate for which the reduction in expected survival from 0.25*K* to *K* was on average 33 % (Fig. 3a). Adult survival also showed a decrease with increasing relative population size (Fig. 3b, average reduction=16%), whereas variation in relative population size only had a small effect on the fecundity rate (Fig. 3c, average change=1.2%).

**Figure 4:**
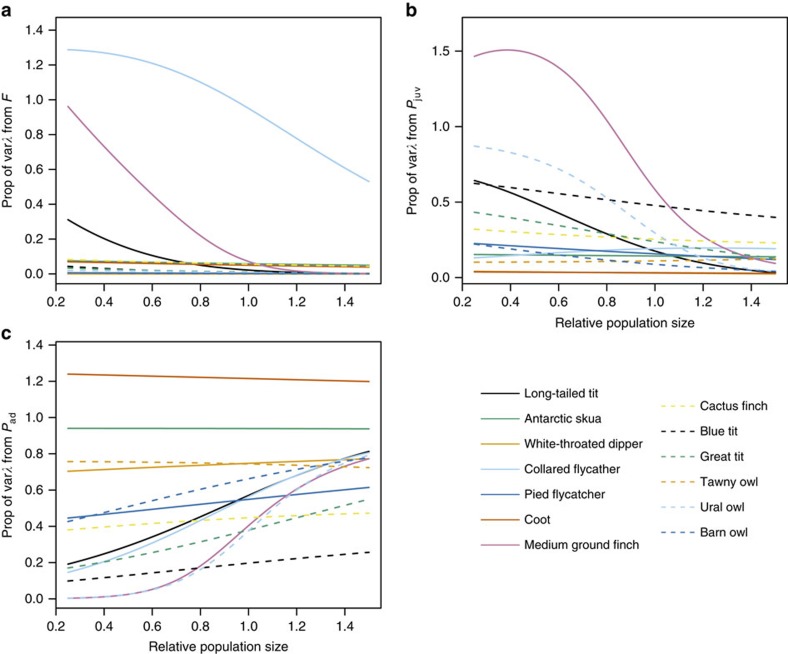
Variance in the different components of the population growth rates *λ* in relation to population size. Interspecific differences (for species identification, see Fig. 2) in the relative contribution of environmental variance in fecundity rate *F* (**a**), juvenile survival rate *P*_juv_ (**b**) and adult survival rate (**c**) to var *λ* as a function of relative population size 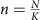 (Fig. 2).

Our final step is to examine how environmental stochasticity in each demographic rate contributes to variation in the population growth rate at different population sizes. For a given population, mean fecundity, juvenile survival and adult survival are determined at a fixed density, but population size fluctuates as a result of environmental stochasticity within each rate and the environmental covariance between rates. We then calculate the proportion of variation in the rate of growth of a population attributable to environmental variation in each rate (Fig. 4). Overall, the contribution of variation in fecundity (Fig. 4a, except in 1 species) and juvenile survival (Fig. 4b, except in 2 species) decreases significantly towards carrying capacity. However, as population size approached carrying capacity, the contribution of variation in the adult survival rate to variation in population growth increased in 11 of the 13 species (Fig. 4c). These patterns reveal that at small population sizes, population dynamics are primarily driven by variation in recruitment, which is strongly influenced by environmental stochasticity (Fig. 1). In contrast, when close to *K*, variation in population growth is more strongly influenced by variation in adult mortality.

## Discussion

Summarizing these patterns, our analyses revealed that a large proportion of interspecific differences in population variability in birds is related to cross-species variation in the influence of environmental stochasticity on the population dynamics (Fig. 1; Supplementary Fig. 2). Environmental stochasticity simultaneously impacts all vital rates, but most strongly affects temporal variation in the number of new recruits entering the population^24^. The magnitude of variation in population size is consequently determined primarily by the amount of environmental stochasticity a population experiences rather than by the strength of density-dependence. A consequence of this is that the return time to equilibrium, which expresses the strength of density-dependence^23^, was independent of variation in population size around *K*. Second, regulation of population size was most strongly influenced by density-dependence in survival (Fig. 2). In contrast, in spite of large annual fluctuations in fledgling production in many of the species included in this study, density-dependence in fecundity explained only a small proportion of variation in the strength of density-dependence (Fig. 3c, see also Supplementary Fig. 2 for the loglinear model of density regulation).

These patterns help us to classify avian population dynamics based on the relative contributions of environmental stochasticity and density-dependence to fluctuations in population size. At one extreme, we observe dynamics characterized by recruitment-driven fluctuations caused by pulsed-resource dynamics^25^. This kind of dynamic occurs when the resources available for reproduction or survival 1 year are independent of availability of resources in previous years. Fluctuations in resource availability generate dynamics characterized by a large contribution of environmental stochasticity and strong density-dependence with populations tending to collapse in size following years of high resource availability^26^,^27^. The population dynamics of the Galapagos-finches and the three owl species provide good examples of this class of dynamics. Specifically, the production of new recruits occurs in years with super-abundant food resources (seeds or voles), but is quickly followed by strong density-dependent (primarily juvenile) mortality in years when resources are scarce^21^,^28^,^29^. In contrast to this dynamic, another group of species exhibit relatively smaller fluctuations in population size (Fig. 1), operating primarily through access to breeding sites^21^,^30^ . This stability is a result of strong density-dependent regulation, with excess individuals that do not secure a territory or nesting site much more likely to die compared to those individuals that hold one. Antarctic skua *Stercocarius maccormicki* and blue tit *Cyanistes caeruleus* provide examples from this end of the continuum.

In conclusion, these analyses show that environmental stochasticity rather than variation in the strength of density-dependence is the major factor affecting interspecific differences in population variability of bird populations. Our analyses also reveal a sequence of changes in different demographic rates as the carrying capacity *K* is approached^3^,^30^. As expected from the Lack's^4^ hypothesis, recruitment changes with variation in population size, but has a reduced influence compared to adult survival on population variability around *K*. Thus, the Lack's simple conceptual model^17^ explaining regulation and limitation of bird populations can consequently provide a general framework for quantitative analyses of vertebrate population dynamics in seasonal environments.

## Methods

### Study populations

The population of Antarctic skua *Catharacta maccormicki* was studied at Terre Adélie, Antarctica during the period 1984–2004. This is a highly territorial species that lays on average ∼1.5 eggs^31^. The females start to breed from the age of 4 years for then to breed once every year^32^. The conspicuous life style of this species make the detection probability extremely high (1 and 0.56 for breeding adults and non-breeders, respectively)^33^.

Barn owls *Tyto alba* included in this study were captured and ringed in the Payerne region in western Switzerland during the period 1993–2010, where they mainly breed in nest boxes (see Altwegg *et al*.^34^ for further details about field procedures). The probability of breeding by the females as 1 year old is about 0.7 (ref. 35) . The average clutch size is ∼ 6 eggs^35^. The fluctuations in population size in this area are strongly influenced by severe winter weather causing large mortality^36^. The probability of an adult to be recaptured alive was >0.8 in this study population^34^.

Blue tits *C. caeruleus* were studied at Vlieland, which is an island in the Dutch Waddensea, and consists of mixed pine-deciduous woodland. Here almost all Blue Tits breed in nest boxes, resulting in very precise population counts. When the nestlings were 7–10 days old, the parents were caught on the nest using a spring trap. The number of fledglings was counted and ringed at day 10-15 after hatching.

The collared flycatcher *F. albicollis* is a small migratory hole-nesting passerine^37^. Data on collared flycatcher were collected in several study plots at the island of Gotland (57° 30′ N 18° 33′ E), Sweden, during the period 1986–2007 as part of a long-term study of individual-based demography and genetic, and phenotypic variation in several fitness-related traits^38^,^39^,^40^,^41^. A large proportion of all breeding individuals were in each year caught in nest boxes, resulting in recapture rates of non-dispersers higher than 0.97 (ref. 40). Individuals subject to experimental manipulations were excluded from the analyses.

The cactus finch *Geospiza scandens* and medium ground finch *G. fortis* belong to the ground finch group of Darwin's finches, with the cactus finch being the larger species (∼20 g) than the medium ground finch (16 g)^26^. The data were collected at Isla Daphne Major, Galápagos, Ecuador during the period 1979–1998. Attempts were made to find every nest and identify the parents in every year of breeding. Nestlings were colour-ringed in the nest at the age of 8 days^42^. In this system, prolonged rainfall associated with El Niño events has a strong influence on the population dynamics of Darwin's finches, with production of large cohorts of offspring, for example, in the years 1983, 1987 and 1991 (ref. 43). In contrast, no fledglings were produced in drought years. There was a large individual variation in fitness contributions to the future generations in both species, especially influenced by a few individuals that live for an exceptional long time, enabling a large number of breeding attempts^42^,^44^. The standard errors of the population estimates of these two species were small (for the medium ground finch, see Fig. 56a in Grant^26^) because between 1980 and 1992 a large proportion (>90 %) of all individuals were individually colour-ringed, facilitated by the small size of island (0.34 ha)^43^.

The coot *Fulica atra* is a precocial waterbird that is highly territorial during the breeding season. This species was studied at the lake Westeinderplassen (57° 18′ N 4° 42′E), The Netherlands, during the period 1966–1988. In this area most adults are marked with steel leg bands and numbered plastic neck collars^45^. Throughout the breeding season the study area was searched for nests and the number of surviving chicks counted. The chicks were ringed from 1 week to 4 weeks after hatching^45^. Experimental evidence indicate that fledgling success are strongly dependent on the availability of food during the breeding season^46^. The recapture probability was high in both males (0.97) and females (0.76), most likely caused by a higher probability of females skipping a breeding season than males^47^.

The study population of the white-throated dipper *Cinclus cinclus* was located in the Lygndalsvassdraget along the river Lygna in the county of Vest-Agder in southern Norway (58° 15′ N 7° 15′ E). The study area covers ∼60 km from the mouth of the river towards the inland. The population size was estimated during the period 1978–2012 by checking appropriate nesting sites along the banks of the river for active nests throughout the breeding season. By mistnetting and ringing of nestlings, a large fraction of the breeding adults was individually colour-ringed. Because of the large proportion of all individuals ringed and the conspicuous behaviour of the adults especially in the mornings during the breeding season, the bias in population estimate is likely to be small and recapture rates were high^48^. The population fluctuations are driven by a combination of density-dependence and environmental stochasticity caused by icing of the river during cold spells in the winter^48^,^49^.

Great tits *Parus major* were studied in a Hoge Veluwe, the Netherlands (52° 23′ N 05° 51′ E), where a large proportion of all individuals nest in nest boxes. Recapture probability was very high in females (average=98.7%)^50^. The forest is mixed coniferous–deciduous woodland and included until a storm in 1972 a large block of pure pine plantation. In all areas nest boxes were visited at least once every week. The number of females present in an area in a given year was defined as the number of first clutches. The number of fledglings produced was determined by the number of nestlings present at day 15. The dynamics of this population was strongly influenced by the number of immigrants from surrounding areas^51^.

The population dynamics of long-tailed tits *Aegithalos caudatus* was studied using uniquely colour-ringed individuals in the Rivelin Valley, South Yorkshire, UK (53° 23′ N 1° 34′ W) during the period 1995–2013, but excluding 2001 when parts of the study area were not accessible. The long-tailed tit is a small (≈8 g) cooperatively breeding passerine that builds a domed nest and lay a large clutch (typically 9–11 eggs). Any unringed breeder was caught by mist-nets. Nestlings were counted and individually marked at day 11 of the nestling period. Adults and nestlings were sexed using molecular techniques^52^,^53^,^54^. The annual recapture probability of adults was consistently high both for males (0.92) and females (0.83)^54^.

Pied flycatchers *Ficedula hypoleuca* were studied by an extensive use of nest boxes in the forest of Hoge Veluwe, the Netherlands (see above), where it first was recorded breeding in 1959. After that year, the population increased rapidly until it reached a period with stationary fluctuations around a carrying capacity of 86 pairs^55^. Almost all pairs bred in nest boxes. Data on reproduction and survival were obtained through weekly inspection of nest boxes, and banding of chicks and adults with uniquely numbered aluminium rings.

The analyses of the population dynamics of tawny owl *Strix aluco* and ural owl *Strix uralensis* were based on data collected from birds breeding in nest boxes in two Finnish study sites approximately 150 km apart^56^. Females were captured during incubation and ringed if not already banded as a nestling. The tawny owl population was studied in Kirkkonummi (60° 13′ N 24°15′ E) during the period 1978–2008. The population size was estimated based on the number of inhabited nest boxes in addition to the number birds actively responding to playback in territories where the pair was assumed to breed in natural cavities. Thus, the population estimates were likely to be precise. The recapture probability varied with the rodent cycle, and was highest after the increase phase (average 0.78)^29^. The ural owl population was located in Heinola (60° 13′ N 24° 15′ E) and data only from individually known females breeding in nest boxes during the period 1977–2008 were included. On average, 35 % of the breeding females were ringed as nestling in this population^56^. A territory was considered occupied if eggs or offspring were produced in the nestbox, the female was actively guarding an empty nestbox or scrapings in the sawdust that covers the bottom of nestbox were found during the breeding season^57^.

The time series of fluctuations in population size as well as temporal variation in the different vital rates are summarized in Supplementary Fig. 1.

### Population model

All time series included in the present study show density-dependence, characterized as a negative relationship between changes in population size from year *t* to *t*+1 and population size in year *t.* Estimating the form of the density regulation in natural populations is notoriously difficult^58^,^59^. We therefore characterize the population dynamics of each species by fitting a logistic model to the time series of fluctuations in *N*^7^, so that the change in population size is:


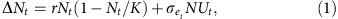


where *N*_*t*_ is the population size at time *t*, 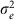 is the environmental variance, *r* is the intrinsic growth rate, *K* is the carrying capacity and *U*_*t*_ is a sequence of independent variables with mean 0 and variance 1. This model has previously been shown to provide a realistic model of density regulation in many bird populations^60^,^61^,^62^. The diffusion approximation for this model has infinitesimal mean and variance *rN*(1−*N*/*K*) and 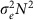, respectively^2^, which gives the variance in the stationary distribution of population sizes


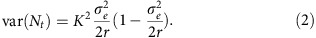


This approach enabled estimation of the carrying capacity *K* as well as the variance of the stationary fluctuations around *K*^2^.

We also modelled the dynamics of the species by the familiar loglinear model of density regulation^27^,^63^. This enabled us to examine whether the relationship between variation in different vital rates and patterns in population dynamics was dependent upon the choice of model for the density regulation. The results of these analyses are presented in the Supplementary Fig. 2; Supplementary Table 1.

Interspecific comparisons were facilitated by standardizing *N* in relation to *K*.

### Matrix model

The population dynamics were modelled with a stochastic density-dependent projection matrix **I**, assuming a post-breeding census^15^. Density-dependence and environmental stochasticity were included in all non-zero elements. We assume that only the last two age classes reproduce with rate *f*, that prior to sexually maturity individuals survive with age-specific probability *p*_1_, and that individuals in the oldest age class survive with probability *p*_2_.

Survival rates of juveniles from fledging until age of sexual maturity and adults were estimated based on a Cormack–Jolly–Seber model^64^. The number of fledglings produced by a sample of females was assumed to be Poisson distributed . The effects of environmental stochasticity in a given year were assumed to be multinormally distributed among vital rates at link scale.

The stochastic density-dependent projection matrix **l** was based on post-breeding census. The age classes were numbered by 1,2,…*k*, *k* ≥2. Only the last two age classes reproduce, so that the element *l*_1,_*j*=0 for *j*=1, 2, …*k*−2, if *k* >2. Individuals in age classes 1, 2, …*k*−1 survive with probability *p*_1_, whereas those in age class *k* survive and remain in this class with probability *p*_2_. Individuals in age classes *k*−1 and *k* produce on average *f* offspring. Our analyses are based on the surviving fledglings up to age at maturity *k*−1, *q*, which gives *p*_1_=*q*^1/(*k*−1)^. The first row of the projection matrix **l** then becomes 0, 0, …, *p*_1_*f*, *p*_2_*f*, the subdiagonal elements are all *p*_1_, while the element *l*_*kk*_=*p*_2_ and all other elements are zero.

Let the stable age distribution and reproductive values for the projection matrix **l** be **u** and ***v*** respectively, so that **lu**=*λ***u** and **vl=***λ***v** with Σ*u*_*i*_**=**1 and Σ*u*_*i*_*v*_*i*_**=**1, where the population growth rate *λ* is the dominant real eigenvalue of **l**^15^. The sensitivities of^15^ *λ* with respect to *p*_1_, *p*_2_ and *f* in this model, using the fact that *dλ*/*dl*_*ij*_=*v*_*i*_*u*_*j*_ are:


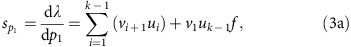



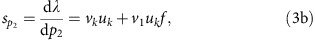


and


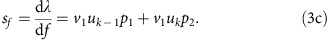


Similarly, the elasticities that express the relative effect of a change in a parameter on *λ* (ref. 15) are:


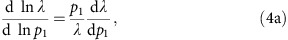



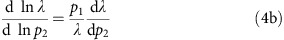


and


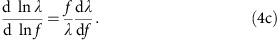


Then the sensitivities and elasticities with respect to *q* are:


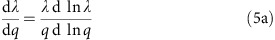


and


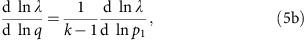


respectively.

### Estimation of parameters

Let *F*_*t*_ fledglings be produced by *N*_*f*_ females in year *t*. Then we assume that


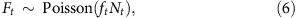


where logit(*f*)=α_*f*_+β_*f*_*N*_*t*_+ɛ_*t*,1_. Here ɛ_*t*,1_ is the environmental variance. The survival from fledgling to recruitment was modelled as


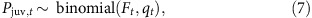


where logit(*q*_*t*_)=*α*_*q*_+*β*_*q*_*N*_*t*_+*ɛ*_*t*,2_

The survival rate of adults *p*_2_, assumed constant after age of maturity, was estimated based on a Cormack–Jolly–Seber model where the capture–recapture data was summarized in a so-called *m*-array^64^,^65^. The cell probabilities of the multinomial likelihood in this model depend jointly on adult survival rate and recapture probability, that is, the probability of capturing an individual is alive^66^. The recapture probability *P*(0) was assumed constant among years and was given a uniform prior distribution *P*(0)**=**uniform(0,1). Annual survival of adults were modelled as


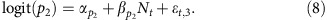


The effects of the environmental conditions in a given year, *ɛ*_*t,k*_, *k*=1, 2 , 3, were assumed multinormally distributed, independent of population size *N*.

Calculating the expected values of *q* and *p*_2_ requires numerical integration since the moments of the logit-normal distribution have no analytical solution^67^. Let the relative population size in year *t* be *n*_*t*_=*N*_*t*_/*K*, where *K* is the carrying capacity estimated from the time series analysis using a logistic model of density regulation^2^. For a given *n*_1_ and estimated parameters we obtain normal distributions at the logit scale with expectations *α*_q_+*β*_*q*_*n*_1_ and *α*_*p*2_+*β*_*p*2_*n*_1_ and corresponding variances 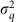 and 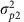, respectively. The expectations E(*q*|*n*_1_) and E(*p*_2_|*n*_1_) can then be found by numerical integration.

Assuming an equal sex ratio among the fledglings, the expected value of female offspring given a certain population size *N*_2_ is E(*F*|*n*_2_) is 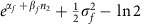.

The strength of density-dependence *d*_*λ*_/*d*_*n*_ can be estimated from equations (1)–(3) and partitioned into components from the fecundity rate, the juvenile survival rate and adult survival rate so that


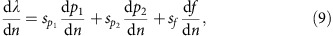


where 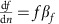, 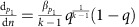 and 
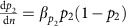
.

The annual variation in population size as well as in the estimates of the three demographic rates is shown for each species in the Supplementary Fig. 2.

### Data availability

The data that support the findings of this study are available from the corresponding author (B.-E.S.) upon request.

## Additional information

**How to cite this article:** Sæther, B.-E. *et al*. Demographic routes to variability and regulation in bird populations. *Nat. Commun.* 7:12001 doi: 10.1038/ncomms12001 (2016).

## Supplementary Material

Supplementary Information Supplementary Figures 1-4, Supplementary Table 1 and Supplementary Note 1
