# Supplementary material for: Demographic routes to variability and regulation in bird populations
Source: Nat Commun. 2016 Jun 22;7:12001. doi: 10.1038/ncomms12001 (PMC4917965; doi:10.1038/ncomms12001)
Supplement: Supplementary Information — Supplementary Figures 1-4, Supplementary Table 1 and Supplementary Note 1 [file ncomms12001-s1.pdf]

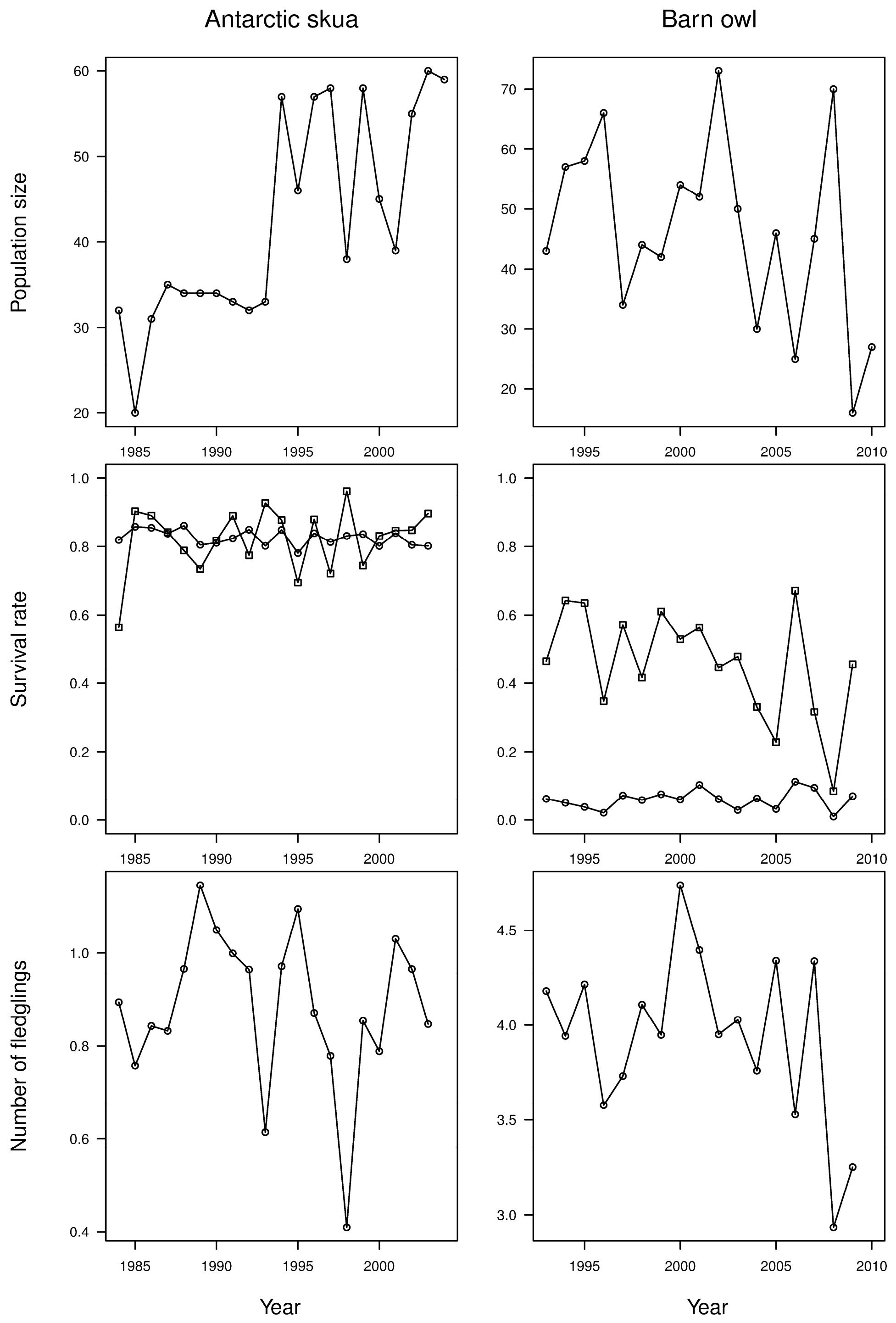

Antarctic skua
Barn owl
Population size
Survival rate
Number of fledglings
Year
Year

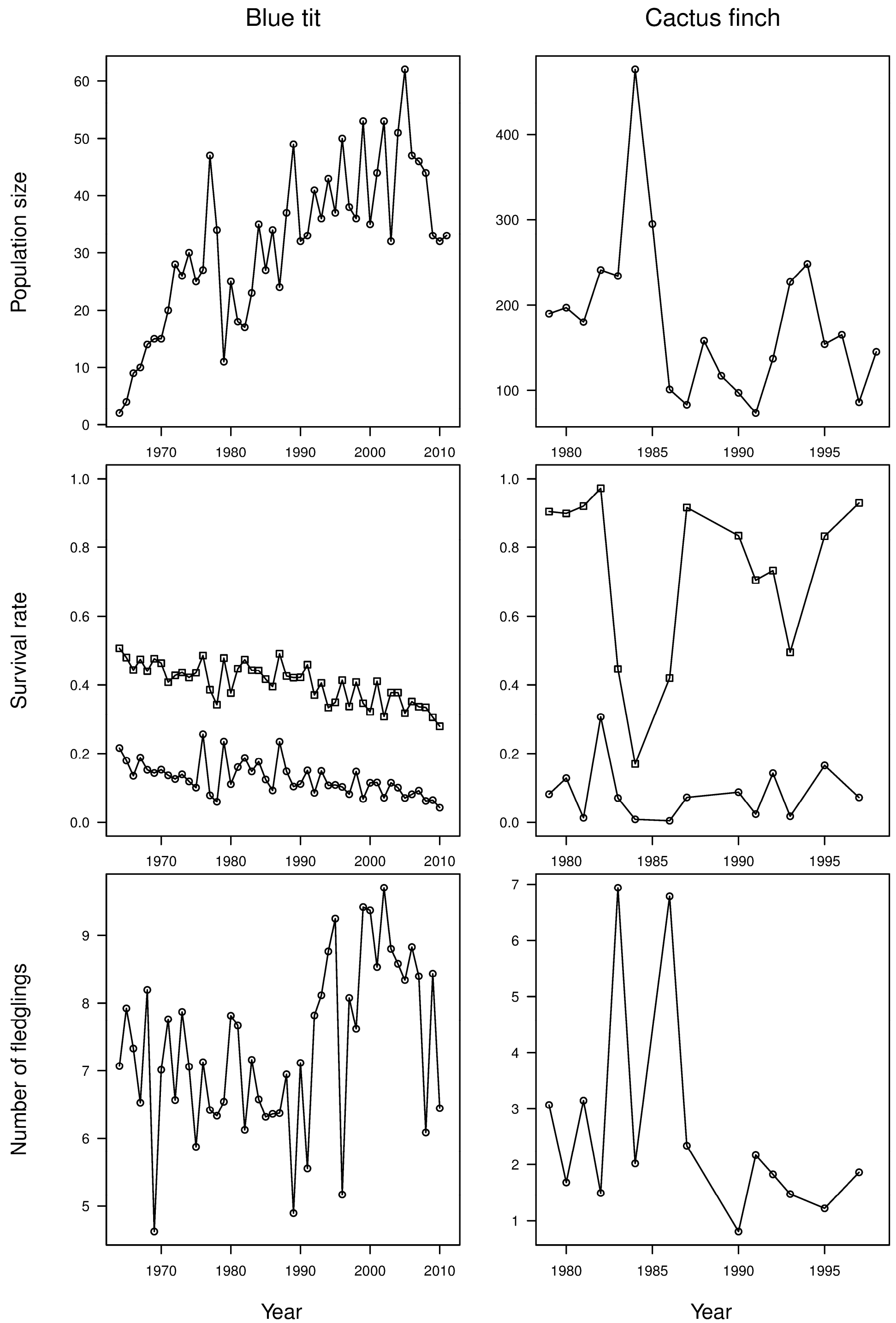

Blue tit
Cactus finch
Population size
Survival rate
Number of fledglings
Year
Year

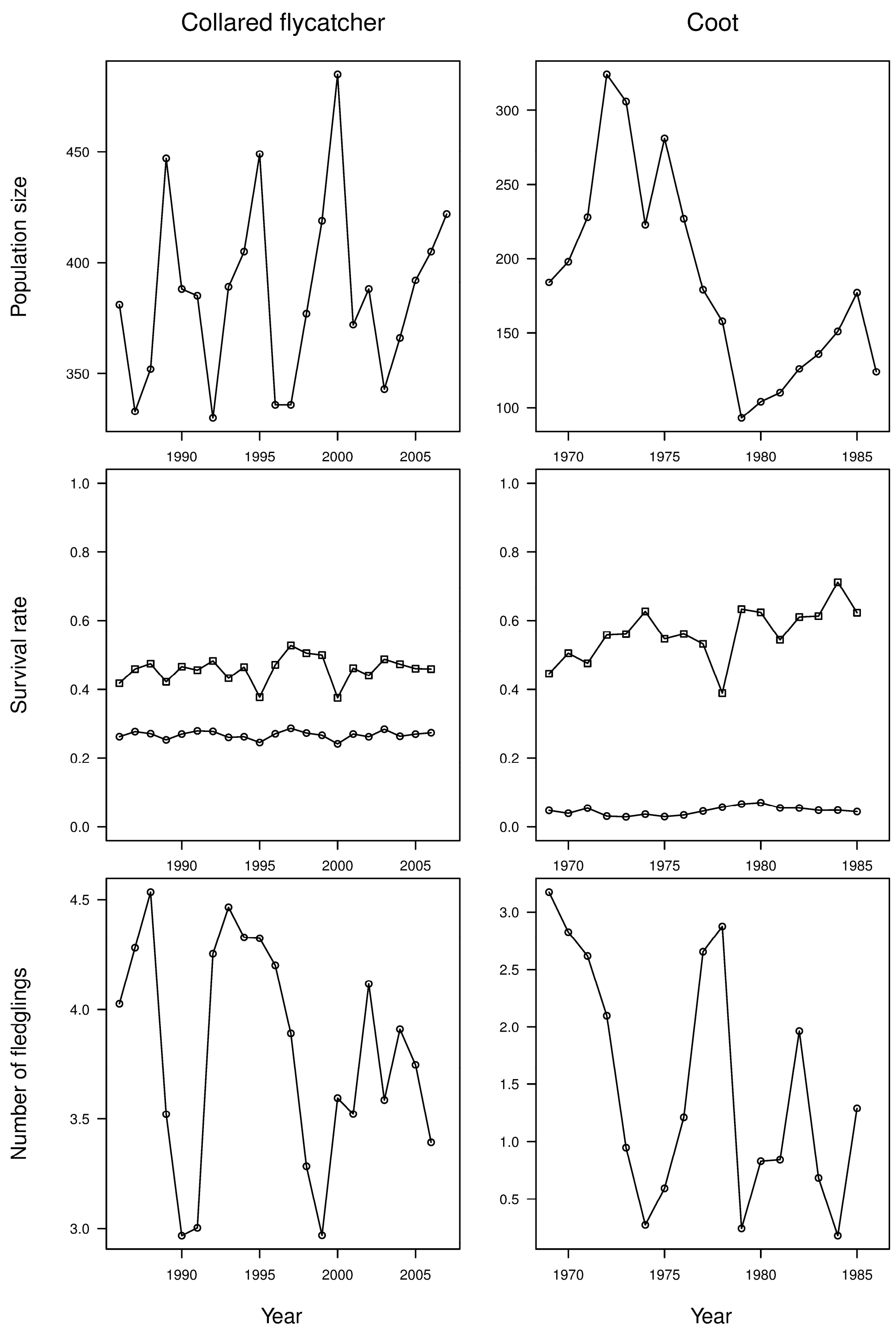

Collared flycatcher
Coot
Population size
Survival rate
Number of fledglings
Year
Year

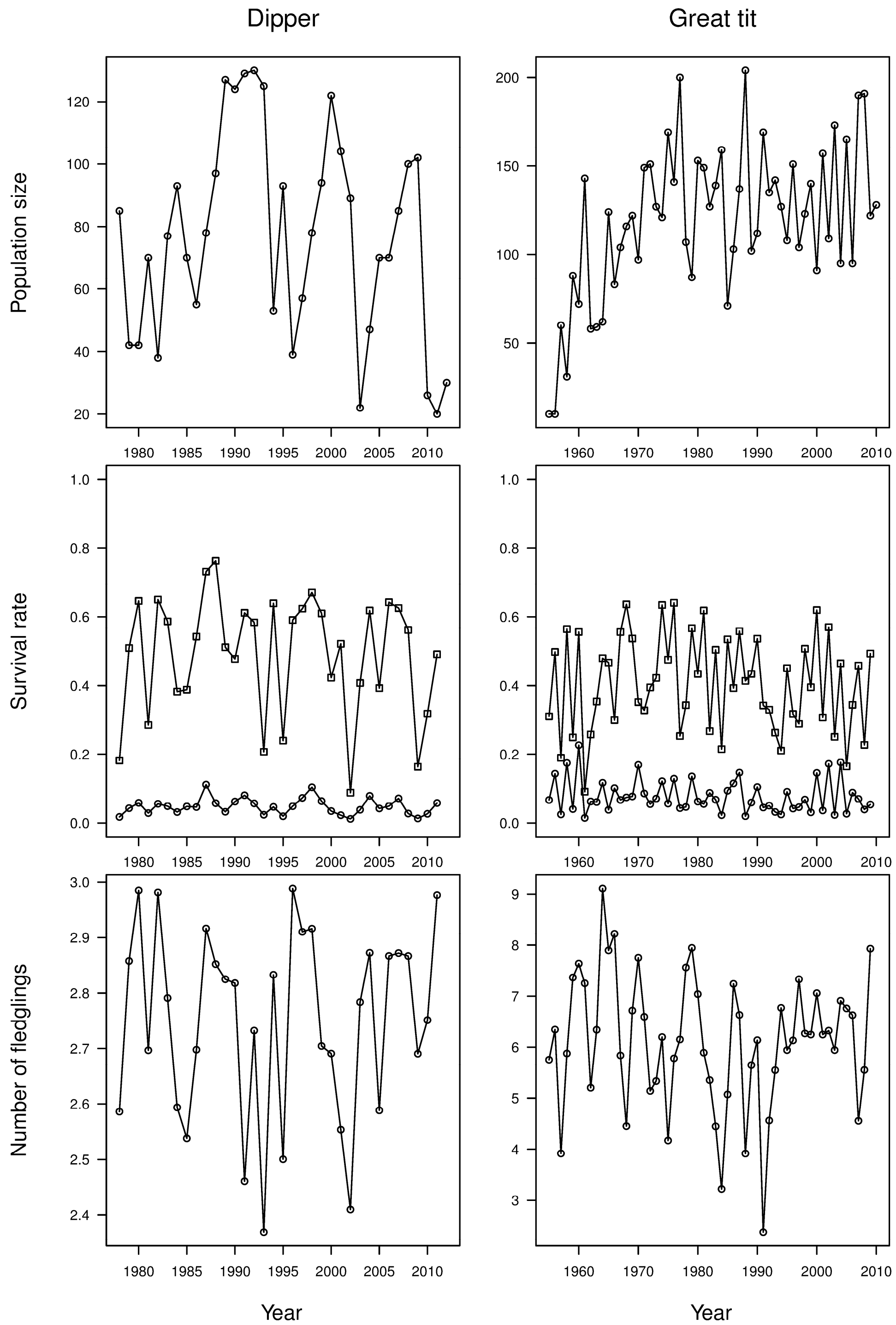

Dipper
Great tit
Population size
Survival rate
Number of fledglings
Year
Year

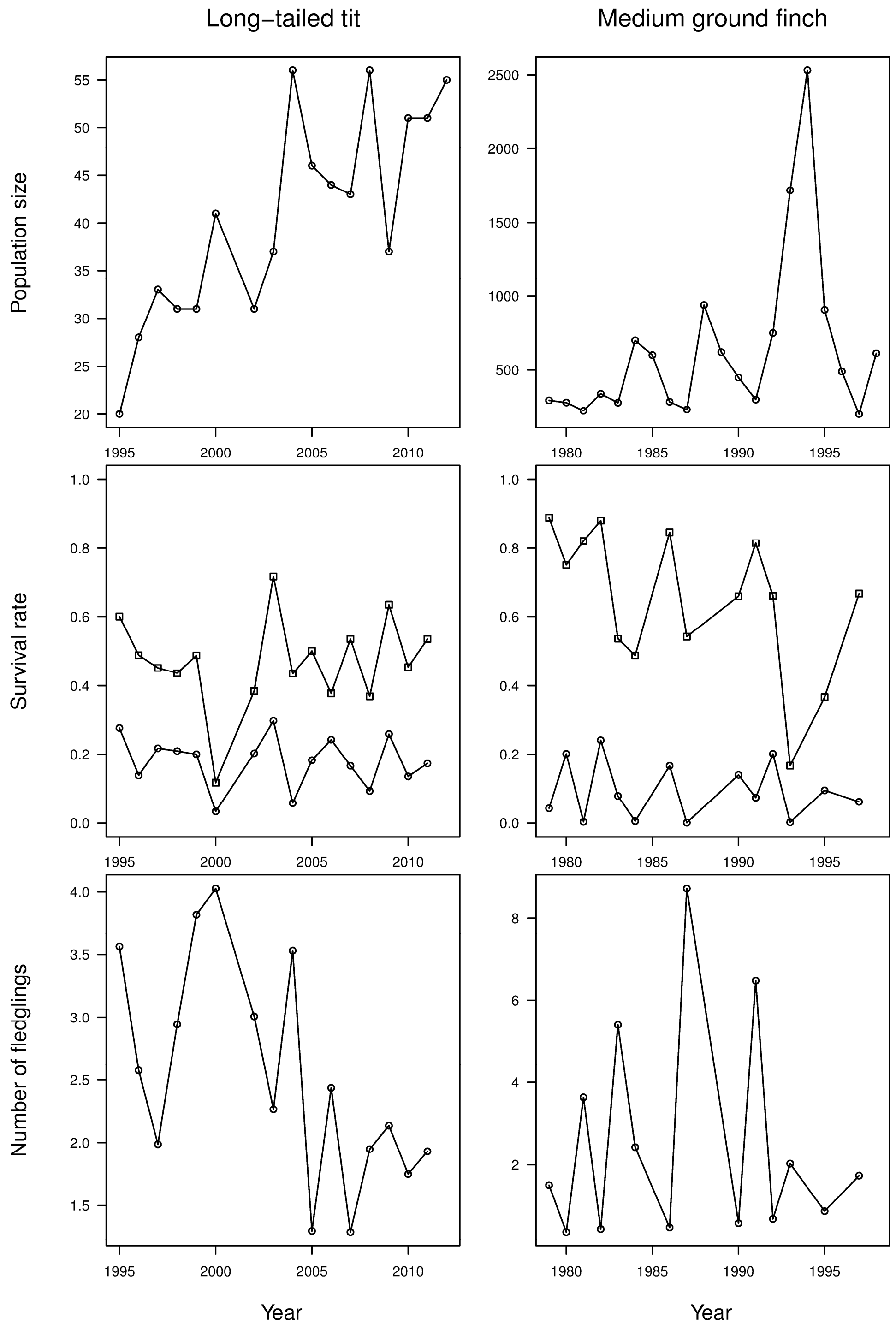
Long−tailed tit
Medium ground finch
Population size
Survival rate
Number of fledglings
Year
Year

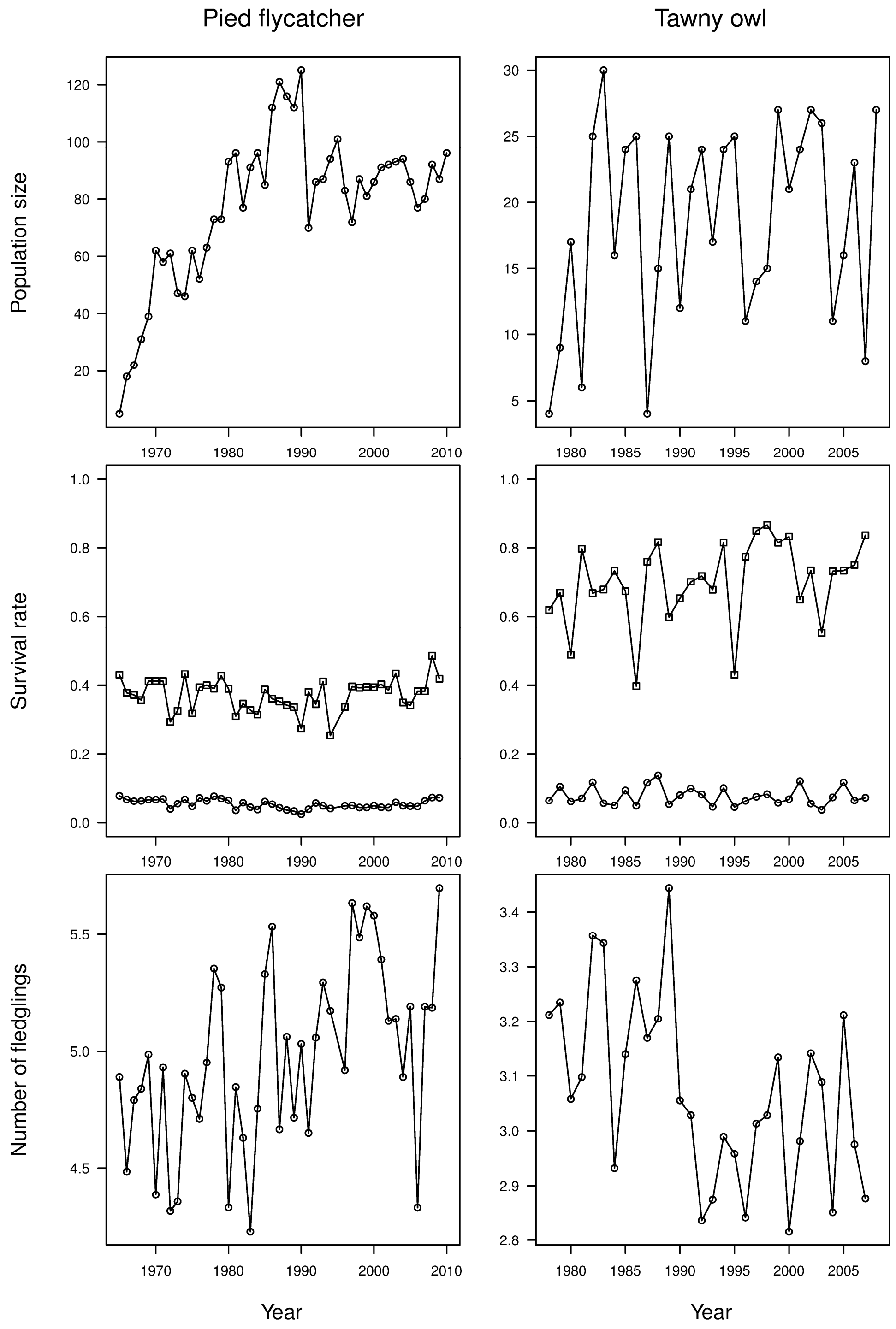

Pied flycatcher
Tawny owl
Population size
Survival rate
Number of fledglings
Year
Year

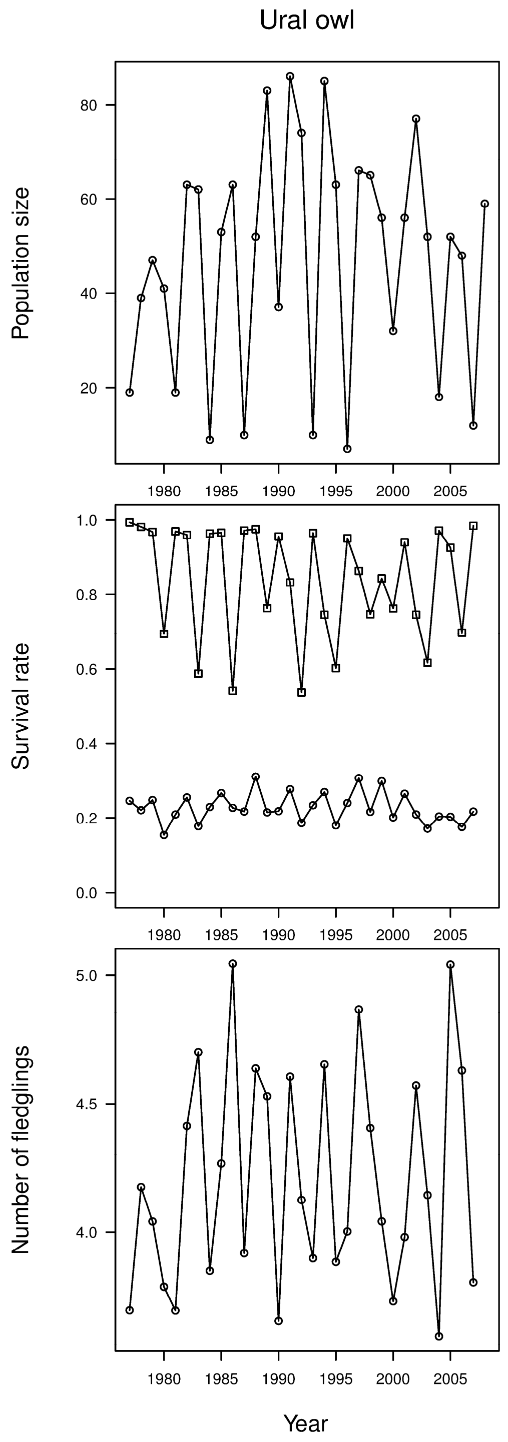
Ural owl
Population size
80
60
40
20
Survival rate
1.0
0.8
0.6
0.4
0.2
0.0
Number of fledglings
5.0
4.5
4.0
1980
1985
1990
1995
2000
2005
Year

Supplementary Figure 1| Temporal variation in population size and demographic traits. Fluctuations in population size (number of breeding females during the study period (upper graph), annual variation in juvenile (circles) and adult (squares) survival rate (middle graph) and in the mean number of fledglings produced per breeding female (lower graph) for the different species. In the estimates of juvenile survival rate we have assumed that the probability of survival was age-independent prior to age of maturity,

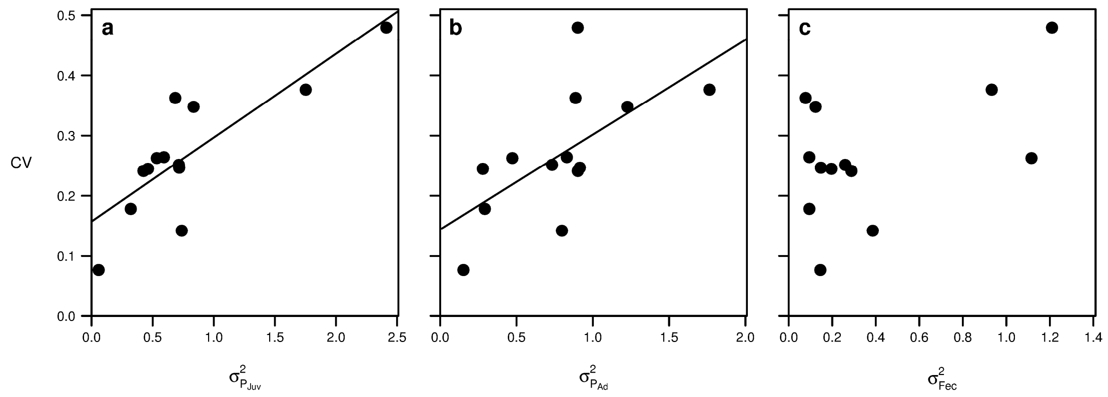

Supplementary Figure 2 | Interspecific differences in population variability in relation to environmental stochasticity in different vital rates. The coefficient of variation in the stationary distribution of population sizes around the carrying capacity *K*, assuming a loglinear model of density regulation, in relation to environmental stochasticity in juvenile survival rate (a), adult survival rate (b) and number of fledglings produced (c). The equations for the regression lines are y = 0.14x+0.16; r^2=0.68; df=1, 11; P <0.001; y = 0.16x+0.14; r^2=0.41; df=1, 11; P < 0.01; and y = 0.13x+0.22; r^2=0.26; df=1,11; P =0.073 for juvenile survival rate, adult survival rate and fledgling production, respectively.

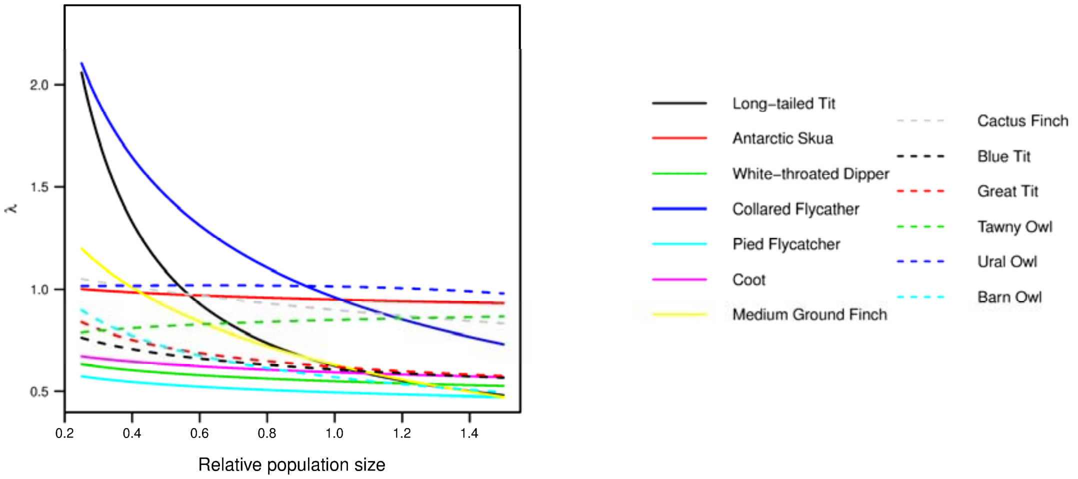

Supplementary Figure 3 | Interspecific differences in the density dependence of the population growth rate λ. The change with population size $n = \frac{N}{K}$, (where the carrying capacity *K* was estimated from the time series of populations fluctuations using a loglinear model of density regulation) against the population growth rate λ calculated at different relative population sizes *n*.

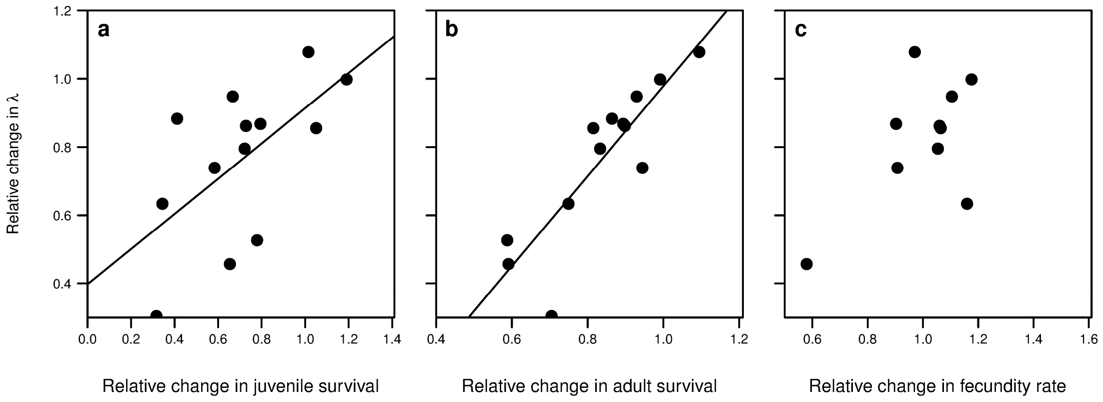

Supplementary Figure 4| Influence of different vital rates on the density dependence in the population growth rate λ. Interspecific differences in the relative change of λ from 0.25*K* to *K* in relation to relative changes in juvenile survival rate (a), adult survival rate (b) and fecundity rate (c) over the same interval of variation in population size using the loglinear model of density regulation. The equations for the regression lines are $y = 0.51x+0.40$; r^2=0.37; df=1, 11; P =0.03; $y = 1.32x-0.35$; r^2=0.76; df=1, 11; $P < 0.001$; and $y = 0.15x+0.60$; r^2=0.20; df=1, 11; P =0.12 (not shown) for juvenile survival rate, adult survival rate and fecundity rate, respectively

Supplementary Table 1. Interspecific variation in the deviance for fitting a logistic and a loglinear model of density regulation.

Species	Logistic model	Loglinear model
Antarctic skua	1711.29	1710.95
Barn owl	406.83	406.16
Blue tit	979.69	979.88
Cactus finch	400.49	400.52
Collared flycatcher	744.33	743.98
Coot	416.73	416.57
Dipper	814.69	814.20
Great tit	1435.83	1434.09
Long-tailed tit	321.15	321.04
Medium ground finch	410.95	410.81
Pied flycatcher	1096.50	1097.16
Tawny owl	656.57	656.24
Ural owl	1093.65	1093.98

Supplementary Note 1

Analyses of a loglinear model of density regulation

To examine whether the interspecific variation in avian density dependent demography depended on the choice of model for the density regulation we also fitted a loglinear model in which variation in demographic traits was related to variation in $\ln N$. The model of density regulation had small influence on the deviance (Supplementary Table 1), indicating that both models described variation over the range of variation in population sizes included in the present data set equally well.

The patterns appearing from the analyses of the logistic model also were evident using a loglinear model of density regulation. The positive associations between the CV and environmental stochasticity in juvenile survival (Supplementary Figure 2a), adult survival (Supplementary Figure S2b) and fecundity rates (Supplementary Figure 2c) were similar to the ones appearing for the logistic model. Furthermore, there was considerable interspecific variation in the shape of density-dependence (Supplementary Figure 3). As for the logistic model of the density regulation, in all but one species (Tawny owl) the population growth rate decreased as population size increased (Supplementary Figure 3). The exception provided by the Tawny Owl may be affected by a relative small carrying capacity (Supplementary Figure 1)., causing a reduction of the growth rate due to the effects of demographic also around *K*.

The regression of the relative difference in the population growth rate against the relative change in each demographic rate showed also for the loglinear model that there were statistically significant positive associations for juvenile and adult survival, but not for fecundity (Supplementary Figure 4). The most pronounced density dependent changes occurred in juvenile survival rate, for which the reduction in expected survival from 0.25*K* to *K* was on average 29 % (Supplementary Figure 4a). Adult survival also showed a decrease with increasing relative population size (Supplementary Figure 4b, average reduction = 16

%), whereas variation in relative population size only had a small effect on the fecundity rate (Supplementary Figure 4c, average increase = 7.0 %). This was similar to the pattern recorded assuming a logistic model of density regulation.
